# A mixed methods study exploring barriers and facilitators to secondary-care nurses discussing smoking cessation with patients: phase 1 of the Think Quit Study

**DOI:** 10.1186/s12912-025-03597-6

**Published:** 2025-08-05

**Authors:** Megan Elliott, Katy-May Price, Rachael M. Hewitt, Nicky Knowles, Siva Ganesh, Jessica Baillie, Lauren Jones

**Affiliations:** 1 Research & Development Department, Clinical Research Centre, Cwm Taf Morgannwg University Health Board, Royal Glamorgan Hospital, Ynysmaerdy, CF72 8XR UK; 2 Welsh Blood Service, Talbot Green, CF72 9WB UK; 3 School of Healthcare Sciences, Cardiff University, Heath Park, Cardiff, CF14 4XN UK; 4 Public Health Wales, 2 Tyndall Street, Cardiff, CF10 4BZ UK; 5 Independent Statistician, Aberdare, UK; 6 University of South Wales, Treforest, Pontypridd, CF37 1DL UK

**Keywords:** Behavioural science, Framework analysis, Nursing, Public health, Smoking, Smoking cessation, Tobacco

## Abstract

**Background:**

Tobacco smokers have increased hospital admissions and experience more health complications compared to those who do not smoke. As such, admission to hospital presents an opportunity for intervention. Nurses play a fundamental role in health promotion, and are well positioned to implement and deliver smoking cessation interventions, but barriers exist. The present study aimed to systematically identify barriers and facilitators experienced by nurses when undertaking two target behaviours; (1) discussing smoking with patients and (2) referring them to smoking cessation services.

**Methods:**

A convergent mixed methods study was conducted with secondary care nurses in a University Health Board in South Wales. Informed by the Theoretical Domains Framework (TDF) and the COM-B model of behaviour, data were collected using a self-administered anonymised survey and a series of focus groups and interviews that explored experiences of nurses and determinants of the target behaviours. Descriptive and inferential statistics were used to analyse survey responses and explore relationships between nurse characteristics and behavioural determinants. Deductive framework analysis was used to code qualitative data to TDF domains.

**Results:**

Survey responses were received from 110 nurses working across 13 speciality areas. Nurse respondents perceived the target behaviours to be very important, and reported frequently undertaking the target behaviours. From the TDF, skills, social influences, environmental context and resources and reinforcement domains had the lowest scores, indicating greater perceived barriers in these areas. Twenty-one nurses participated in focus groups (*n* = 5) and interviews (*n* = 2). Factors influencing the target behaviours were identified for 10 of 14 TDF domains. Facilitators included seeing these behaviours as consistent with their role and utilising the presence of prompts in the environment, whilst barriers included lack of and inconsistent referral methods, competing priorities and patient resistance.

**Conclusions:**

Nurses recognise the importance of their role to discuss smoking but have limited knowledge of local smoking cessation services and experience barriers in their workspace. Reducing barriers to discussing smoking and referring patients will facilitate nurses to support their patients to achieve the best health outcomes through quitting smoking.

**Supplementary Information:**

The online version contains supplementary material available at 10.1186/s12912-025-03597-6.

## Background

Tobacco smoking is the leading cause of preventable death worldwide, causing an estimated 8 million deaths per annum [1]. Increased daily cigarette consumption is positively associated with risk of smoking-related diseases [2], including coronary heart disease and stroke, cancers of the lung and upper airways, Chronic Obstructive Pulmonary Disease (COPD) and fertility and pregnancy complications [2, 3]. Consequently, rates of hospitalisation are likely to be greater amongst adults who smoke, with 16% of respiratory disease admissions, 8% of cancer admissions and 7% of cardiovascular disease admissions related to smoking [4]. Smokers admitted to hospital have worse outcomes than non-smokers in terms of: increased length of stay, complications and readmissions [5–8], reduced wound healing [9] and immune response [10].

Stopping smoking is the best way to reduce risk of disease incidence and hospitalisation [11] and improve treatment outcomes for patients [12]. Governments across the world have set ambitions for smoke-free generations [13–15] and have established corresponding tobacco control plans [16–18]. Locally, the Welsh Government long-term strategy aims to reduce smoking prevalence from 13% of the population to under 5% by 2030 [19]. Innovative approaches to supporting people to quit and targeting communities with greatest smoking prevalence are essential to realising this ambition. In addition, the current increased demand on secondary care settings may benefit from targeting a reduction in smoking prevalence in the long term through reduced smoking-related hospital admissions.

Existing evidence shows that smoking cessation services that integrate pharmacotherapy and behavioural interventions significantly increase quit rates [20, 21]. In Wales, the NHS Help Me Quit (HMQ) service [22] is an integrated all-Wales smoking cessation service available to support patients to quit, through individual or group structured behavioural support and Nicotine Replacement Therapy (NRT) in primary care, hospital and community settings. Patients can self-refer or be referred by healthcare professionals in these settings. Admission to hospital presents an opportunity to identify people who smok, deliver brief intervention and refer patients to smoking cessation pathways [23, 24]. The National Institute for Health and Care Excellence (NICE) Quality Statement [QS207] states that all people who smoke should receive ‘stop smoking treatment’ on admission to hospital [25]. Healthcare professionals can employ brief intervention techniques and frameworks to structure their support, discuss smoking with patients and make referrals to services [26, 27]. However, they often report barriers to such interventions including lack of time, knowledge, perceived patient motivation and support [28], which may result in low referral rates into services from secondary care settings.

Nurses play a fundamental role in promoting health and preventing illness, through managing care, delivering services and supporting patients to make informed choices about their own health to maximise their quality of life [29]. Nurses are well positioned to implement smoking cessation interventions and refer patients to smoking cessation services [30] by acting as assessors, referrers, educators and practice facilitators [31]. Evidence shows that nurse intervention can increase likelihood of quit success but does not lead to significant differences in outcomes based on intensity of intervention delivery, patient group or hospital setting [32]. Previous qualitative research has identified and grouped 46 factors that impact the implementation and delivery of nurse-led smoking cessation interventions [33] into four conceptual categories: socioeconomic, smoking-related and motivational factors, as well as barriers and facilitators. Further exploration of barriers and facilitators that influence nurses’ ability to implement existing interventions may identify areas where nurses could be empowered and supported, thus improving patient healthcare outcomes related to smoking.

Heterogeneity amongst the current evidence base in terms of the type, style and intensity of smoking cessation interventions, and the role of nurses in their delivery, poses challenges for applying existing findings to specific behaviour change interventions. The ‘Think Quit’ study seeks to contribute to the growing evidence base around the role of nurses in the delivery of smoking cessation interventions in hospital settings. The study aims to co-produce and pilot test an evidence-based intervention, informed by the Behaviour Change Wheel (BCW) [34] that empowers nurses to promote smoking cessation amongst patients in hospital settings. Here, we present the findings from Phase 1 of the Think Quit study, which explored experiences of secondary care nurses, including facilitators of and barriers to two target behaviours; (1) discussing smoking with patients and (2) making referrals to the HMQ smoking cessation service in Wales.

## Methods

### Design and setting

A convergent mixed methods study design was selected, comprising a mixed methods survey to collect generalisable data and semi-structured focus groups and interviews to collect in-depth data, generating complementary data to address the study aim [35]. Therefore, quantitative and qualitative data were collected and analysed concurrently, and were subsequently integrated into a single matrix. The Theoretical Domains Framework (TDF) [36] was used as a framework to identify areas of consensus and convergence regarding the two target behaviours (discussing smoking with patients, and referring them to smoking cessation services). The integrated quantitative and qualitative findings are presented in the discussion. The Good Reporting of A Mixed Methods Study (GRAMMS) guideline was used to prepare this manuscript (Additional File 1) [37].

The study site was a Welsh National Health Service (NHS) University Health Board, which includes three district general hospitals, covering a population of 450,000 people.

### Theoretical frameworks

The Theoretical Domains Framework (TDF) [36] and COM-B model [38] provided a systematic and evidence-based approach to understanding the target behaviours; discussing smoking with patients and making referrals to HMQ where appropriate. The COM-B model conceptualizes behaviour as a function of Capability, Opportunity, and Motivation, providing a comprehensive framework for identifying and addressing determinants of these behaviours. Application of the TDF facilitated a more nuanced exploration of the wide range of factors influencing healthcare professional practice categorised into 14 theoretical domains (see Table 1) and 84 theoretical constructs. These theoretical frameworks informed study design, data collection and analysis. They were integrated with a view to designing an intervention that targets specific determinants of nurses’ behaviour to optimise their approach to smoking cessation and the referral processes. The COM-B model [38] sits at the core of the Behaviour Change Wheel (BCW), which identifies intervention and policy functions, and sets out steps for developing interventions. Future phases of the Think Quit study will follow these steps to co-develop, with nurses, an intervention based on the findings presented here.

**Table 1:** TDF domains, corresponding survey statements, mean survey scores and cronbach’s alpha

TDF Domain	No. of statements	Example statement	Mean (SD)	Cronbach’s alpha
Knowledge	7	I know WHY it is important to talk to patients about smoking and refer them to HMQ in Hospital	3.91 (0.66)	0.820
Skills	3	I have had sufficient TRAINING in discussing smoking and referring to HMQ in Hospital	3.04 (0.51)	0.656
Social/professional role and identity	2	Discussing smoking with patients is part of my JOB ROLE	3.99 (0.92)	0.798
Beliefs about capabilities	3	I feel CONFIDENT in discussing smoking referring patients to HMQ in Hospital	3.37 (0.46)	0.744
Optimism	1	I believe discussing smoking and promoting smoking cessation is/would achieve the BEST OUTCOME for the patients I care for	4.14 (0.77)	-
Beliefs about consequences	3	I am CONFIDENT that patients will engage with HMQ in Hospital if I refer them	3.74 (0.63)	0.584
Reinforcement	1	I have seen lots of EXAMPLES where discussing smoking and referring patients to HMQ in Hospital has resulted in successful outcomes for patients	3.30 (0.97)	-
Intentions	1	I INTEND to discuss smoking and refer patients to HMQ in Hospital in the future	4.06 (0.84)	-
Goals	2	I am able to OVERCOME challenges that might impact by ability to discuss smoking and refer patients to HMQ in Hospital	3.40 (0.82)	0.686
Memory, attention and decision processes	2	I am able to manage COMPETING DEMANDS so that I can discuss smoking and refer patients to HMQ in Hospital	3.40 (0.95)	0.611
Environmental context and resources	3	There are PROMPTS in place to remind me to discuss smoking and refer patients to HMQ in Hospital	3.28 (0.89)	0.760
Social influences	3	I have sufficient SOCIAL SUPPORT from colleagues and leaders to discuss smoking and refer patients to HMQ in Hospital	3.19 (0.47)	0.528
Emotion	1	I am WORRIED about upsetting patients by discussing smoking and referring them to HMQ in Hospital	3.66 (0.96)	-
Behavioural regulation	2	I have ways of MONITORING and RECORDING discussions about smoking and referrals to HMQ in Hospital	3.47 (0.90)	0.649

### Participants and recruitment

Nurses working in secondary care settings in both patient and non-patient facing roles in the participating Health Board were eligible to participate. Convenience and snowball sampling techniques were used to invite participants to complete the survey and/or take part in focus groups. Nurses were recruited through electronic adverts and posters disseminated via Health Board wide channels, senior nursing staff and across hospital sites. Face-to-face study promotion was undertaken via nurse gatekeepers, such as clinical nurse specialists who were part of the wider project team, during ward rounds and promotional stands within the hospital sites. Recruitment to the survey and focus groups was open between July and October 2024. Participants were provided with a Participant Information Sheet and completed a written or electronic Informed Consent Form prior to data collection. Focus groups participants received a £15 voucher as a token of appreciation for their time.

### Data collection

#### Survey

Nurses were invited to complete a self-administered anonymised survey that took up to 10 min to complete (virtual or paper) (see Additional File 2). The survey was developed by the study team and pilot tested by the study advisory group, which included secondary care nurses (*n* = 4), public health specialists (*n* = 5), methodologists (*n* = 3), and patient representatives (*n* = 2). This piloting was to ensure content validity [39] and the data were not included in the final analysis. Fixed-response questions were used to collect personal and role characteristics about the nurse; speciality area, years of experience, pay band, turnover of patients, hospital base, ethnicity, smoking status, training experience and perceived importance of the target behaviours. In order to assess the target behaviours, respondents were asked to self-report the frequency of undertaking six smoking cessation promotion behaviours with patients (see Table 2), using a five-point Likert scale (1 = ‘Never’ to 5 = ‘Always).

The research team developed a questionnaire to assess determinants of the target behaviours using the 14 TDF domains [36]. Thirty-four statements (*n* = 34) were developed, with between one and seven statements relating to each domain (see Table 1). Statements were ordered randomly and a five-point Likert scale (1 = ‘strongly disagree’ to 5 = ‘strongly agree’) was used to assess respondent agreement with each statement. To avoid acquiescence bias, four statements were negatively phrased in the survey.

The survey concluded with open-ended questions asking respondents to list the top five barriers to the target behaviours, five ideas or strategies to overcome these barriers and suggestions for what would support them to undertake the behaviours routinely.

The online version of the survey required respondents to complete all questions before proceeding to the next section, and all paper surveys contained complete data. Monitoring of the consent forms by the research team revealed no duplicates.

#### Focus groups

The semi-structured focus group topic guide explored factors relevant to the target behaviours, informed by the TDF and COM-B model [36, 38, 40] (see Additional File 2), and was developed by the team with expertise in qualitative research, public health, health psychology and nursing. Participants were also asked for suggestions of interventions and additional support that may facilitate these behaviours in the future.

Focus groups (face-to-face or virtual) were led by two facilitators (ME, KMP or RH). Open discussion between participants was encouraged to elicit natural conversations and allow for nurses in different specialities and roles to compare and contrast their experiences. Focus groups were audio recorded, transcribed verbatim and anonymised by removing any identifiable information. While participant validation on the transcripts was considered, ultimately the ethical and practical challenges of this practice in focus group research were recognised [41]. To promote the rigour of the study [42], fieldnotes were documented for each focus group. The team used a standard approach across focus groups, which encompassed practical details (time/date/attendance/facilitators), participant characteristics (hospital site/specialities/variation in the sample), researcher reflections on the focus group, and which TDF domains were captured. The fieldnotes were used to aid study management and as context for analysis but were not analysed as data. Data were analysed iteratively, and data collection continued to the point of data saturation [40].

### Data analysis

#### Survey

Statistical analyses were undertaken by SG using R software version 4.4.2 [43] and interpreted by ME and SG. Descriptive statistics were calculated to describe sample characteristics and self-reported frequency of undertaking the target behaviours. Chi-squared tables of independence were used to evaluate significant associations between sample characteristics, with the level of significance set at ≤ 0.05.

Mean TDF domain scores were calculated from responses to each set of domain-specific statements, and internal consistency of questions within domain with two or more statements (*n* = 10) was assessed using Cronbach’s alpha. A Cronbach’s alpha of greater than 0.5 was deemed sufficient for preliminary research [44], and all domains satisfied this requirement. Domains with Cronbach’s alpha between 0.5 and 0.7 were assessed to determine whether removal of individual statements would improve internal consistency, however no sufficient improvements were observed, so all items were retained for analysis. Overall, Cronbach’s alpha for the scale is 0.9497 and mean scores and corresponding Cronbach’s alpha for each domain are presented in Table 1.

Ordinal logistic regression modelling was used to determine whether mean domain scores were predictive of self-reported frequency of undertaking the two target behaviours; asking about smoking status (Ask) and referring to HMQ (Refer). Given that the behaviour data was collected using 5-item Likert scales, data were treated as ordinal and the average TDF domain variables (predictors) are considered as numerical.

Sub-group analyses were undertaken for four variables; years of experience (comparing nurses with over and under 10 years of experience as a registered nurse), smoking status (comparing nurses who had never smoked with those who currently or previously smoked or used e-cigarettes), Agenda for Change Pay Band (comparing nurses working at Band 5, Band 6, Band 7 and Band 8a-d) and training experience (comparing nurses with any training relating to smoking with those with no training experience). Data were compared for these four sub-groups using independent sample t-tests, Wilcoxon tests, Kruskal-Wallis test and/or Analysis of Variance (ANOVA) with Multiple Comparison (pairwise) tests to ascertain whether any of these variables affected domain scores, perceived importance ratings and/or self-reported frequency of behaviours (see Additional File 3).

Free-text responses were analysed by ME and RH using content analysis [45]. Data were coded using the COM-B model [38] for barriers, and the BCW intervention functions [34] for the intervention suggestions.

#### Focus groups

Focus group data were deductively analysed using framework analysis [46, 47], concentrating on determinants of the two target behaviours; nurses discussing smoking with patients and nurses referring patients to the smoking cessation service, HMQ. The coding framework adopted was based on the 14 domains of the TDF (Table 1). Despite the deductive nature of analysis, data that did not fit the coding framework were extracted and reviewed by the study team to confirm it was not relevant to the target behaviours and actors [40]. We adopted a systematic, iterative, transparent and reflexive process to coding, starting with line-by-line coding of transcripts, then indexing and mapping codes to the framework [48]. The first transcript was fully coded independently by two members of the research team (KMP, RH) and reviewed collectively to reach consensus and agreement of coding to the TDF domains. All transcripts were fully coded by KMP and secondary coding (RH) reduced in coverage with 50% of analysis, 20% and then 10% across subsequent transcripts. The researchers met regularly to discuss any discrepancies and resolved these through discussion. Preliminary analysis was presented to the research team (ME, KMP, RH, LJ, NK) for collective review, discussion and development of an agreed matrix which mapped themes to the TDF domains, using Microsoft Excel. TDF domains that were considered most prevalent in the data were those that had high frequency of occurrence and coverage across focus groups.

## Results

### Survey

The survey results encompass respondent characteristics, perceived importance and self-reported frequency of behaviours, TDF data and free-text comments, and each is reported in turn, highlighting barriers and facilitators to discussing smoking and referring to smoking cessation services.

#### Respondent characteristics

A total of 110 survey responses were received from registered nurses working across 13 secondary care nursing speciality areas. Nurse respondents reported high patient turnover, seeing new patients either every day (*n* = 71, 64.5%) or every week (*n* = 25, 22.7%). Most respondents identified as ‘White’ (*n* = 97, 88.1%), had over 10 years’ experience as a nurse (*n* = 72, 65.5%), reported never smoking (*n* = 78, 70.9%), had received some form of training related to smoking cessation (*n* = 79, 71.8%). Responses were distributed across NHS Agenda for Change Pay Bands 5 (*n* = 39, 35.5%), 6 (*n* = 24, 21.8%), 7 (*n* = 37, 33.6%) and 8a-d (*n* = 10, 9.1%). There were no significant associations between characteristics (see Additional File 3), other than a positive association between years of experience and pay band (χ2 (3, 110) = 14.16, *p* = 0.003).

#### Perceived importance and self-reported frequency of behaviours

Respondents perceived asking patients about smoking status (Ask) and referring to smoking cessation services (Refer) as *very important* (*M* = 4.12, *SD* = 0.96). There were no significant differences in perceived importance of the behaviour by years of experience (*p* = 0.613), nurse smoking status (*p* = 0.968), training experience (*p* = 0.209) or Agenda for Change pay band (*p* = 0.457).

Respondents self-reported their frequency of undertaking six behaviours relating to smoking cessation promotion within their role, with higher scores indicating that behaviours are performed more frequently. Nurses most frequently asked patients about smoking status (*M* = 4.02, *SD* = 1.22) and least frequently recorded smoking status using the Wales Nursing Care Record (*M* = 2.94, *SD* = 1.74). An implementation decay was observed between frequencies of asking about smoking status, delivering brief advice and referring to smoking cessation services (see Table 2).

**Table 2:** Self-reported frequency of undertaking smoking cessation promotion behaviours

Behaviour	Mean (SD)
Ask patient about smoking status	4.02 (1.22)
Record smoking status on Wales Nursing Care Record (WNCR)	2.94 (1.74)
Inform patients of smoke-free policy	3.55 (1.39)
Provide brief advice regarding quitting smoking	3.56 (1.23)
Refer patient to Help Me Quit (HMQ)	3.23 (1.33)
Issue Nicotine Replacement Therapy (NRT)	3.32 (1.45)

Nurses with over 10 years of experience were significantly less likely to report recording smoking status, *F* (1,108) = 7.0316, *p* = 0.0092, informing patients of smoke-free policies, *F* (1,108) = 10.2833, *p* = 0.0018 and provide patients with NRT, *F* (1,108) = 4.4086, *p* = 0.0381. Nurses who had received training relating to smoking (*M* = 3.709, *SD* = 0.137) were significantly more likely to report providing patients with brief advice about smoking cessation than those with no training experience (*M* = 3.194, *SD* = 0.218), *F* (1,108) = 4.0121, *p* = 0.0477. Significant differences were observed for recording smoking status, informing about smoking policies and referring to smoking cessation services when comparing nurses working in Band 5 and Band 6 roles with those in Band 7 and Band 8a-d roles (see Additional File 3). There were no significant differences in self-reported frequency of any behaviours based on smoking status (see Additional File 3).

#### TDF data

Mean scores for each TDF domain are presented in Table 1. High scores indicate strong agreement with statements in the domain, whilst low scores indicate strong disagreement. Domains with the greatest mean scores were optimism, intentions and social/professional identity, suggesting that these domains are less likely to represent barriers to the target behaviours and therefore may represent facilitators. Conversely, mean scores were lowest for skills, social influences, environmental context and resources, and reinforcement, suggesting that these domains may represent barriers experienced by nurses when discussing smoking with patients and referring them to cessation services.

Mean scores were not significantly different (*p* > 0.05) for any domains when comparing nurses with under/over 10 years of experience; nurses who have never smoked with current/past smoker and/or e-cigarette user; and nurses working across Agenda for Change pay bands 5 to 8a-d (see Additional File 3). Nurses who self-reported receiving some form of training related to smoking and/or promoting smoking cessation had significantly higher scores for eight TDF domains; knowledge (*p* = 0.0068), skills (*p* < 0.001), beliefs about capabilities (*p* = 0.0173), reinforcement (*p* = 0.0033), goals (*p* = 0.0102), memory, attention and decision processes (*p* = 0.0074), environmental context and resources (*p* = 0.0102) and social influences (*p* = 0.0067), compared with those who reported receiving no training (see Additional File 3). Training therefore may represent a facilitator to nurses discussing smoking and referring to smoking cessation.

Table 3 shows the information related to the odds ratios for each domain in predicting self-reported frequency of two target behaviours; asking patients about smoking status (Ask) and referring patients to HMQ (Refer). Among the 14 domains, social/professional role and identity, reinforcement, and behavioural regulation were significant predictors of both Ask and Refer (*p* < 0.1), whilst memory, attention and decision processes and social influences were only significantly predictive of Ask (*p* < 0.1) and beliefs about consequences was predictive of Refer (*p* < 0.1). The odds ratio shows the probability that an increase in the domain score would increase the expected value of self-reported frequency of the behaviour. For example, the estimated odds ratio of memory, attention and decision processes implies that the odds of giving a higher frequency response to ask is 3.060, as such a participant is over 3 times as likely to give a higher response to Ask than the ‘never’ response.

**Table 3:** Odds ratio for each domain in predicting self-reported frequency target behaviours (ask, refer)

	Ask				Refer			
	Log odds co-efficient	Std error	*P* value	Odds Ratio	Log odds co-efficient	Std error	*P* value	Odds Ratio
Knowledge	0.076	0.515	0.883	1.079	0.470	0.466	0.313	1.601
Skills	0.895	0.683	0.190	2.447	-0.124	0.596	0.835	0.883
Social/professional role and identity	0.779	0.306	0.011*	2.179	0.552	0.295	0.061*	1.738
Beliefs about capabilities	-0.396	0.823	0.630	0.673	0.524	0.700	0.454	1.688
Optimism	-0.457	0.383	0.233	0.633	-0.311	0.358	0.386	0.733
Beliefs about consequences	0.026	0.550	0.963	1.026	-0.834	0.455	0.067*	0.434
Reinforcement	-0.611	0.299	0.041*	0.543	0.905	0.270	0.001*	2.471
Intentions	0.430	0.388	0.269	1.537	0.042	0.346	0.903	1.043
Goals	-0.025	0.437	0.955	0.975	-0.116	0.371	0.754	0.890
Memory, attention and decision processes	1.118	0.436	0.010*	3.060	0.585	0.367	0.111	1.796
Environmental context and resources	-0.140	0.396	0.723	0.869	0.011	0.371	0.977	1.011
Social influences	-1.724	0.831	0.038*	0.178	-0.113	0.658	0.863	0.893
Emotion	0.360	0.248	0.147	1.434	-0.293	0.221	0.185	0.746
Behavioural regulation	0.643	0.387	0.096*	1.902	0.631	0.359	0.079*	1.880

Note: *p < 0.1

#### Free-text responses

Respondents listed 311 barriers to discussing smoking and referring patients to smoking cessation services. Barriers most commonly related to lack of physical opportunity (*n* = 123, 39.5%), such as time constraints, lack of resources, incompatible environment, competing priorities and staffing shortages. Reflective motivation barriers were also common (*n* = 70, 22.5%), these involved nurses’ evaluations or beliefs about patients not being ready, willing or well enough to engage in conversations and referrals. Other reported barriers included; psychological capability (*n* = 40, 12.9%) regarding lack of knowledge around support available and how to refer; social opportunity *(n* = 40, 12.9%) regarding interpersonal influences and reactions from patients and automatic motivation (*n* = 14, 4.5%) regarding emotional responses, particularly stress and anxiety. Minimal data was coded to physical skill (*n* = 6, 1.9%) and 17 responses (5.5%) had insufficient information to be coded.

Intervention ideas and strategies (*n* = 273) to facilitate smoking discussions and referral to smoking cessation services most commonly aligned with the environmental restructuring (*n* = 116, 42.5%), training (*n* = 34, 12.5%) and education (*n* = 26, 9.5%) intervention functions. Minimal data was coded to modelling (*n* = 9, 3.3%), enablement (*n* = 7, 2.5%), persuasion (*n* = 2, 0.7%) and incentivisation (*n* = 1, 0.4%), no data were coded to coercion and restriction and 77 responses were not coded as they were not related to the target behaviours (*n* = 36, 13.2%) or had insufficient detail (*n* = 26, 9.5%). Suggestions offered included; simplification of the referral system, new methods to refer to smoking cessation, education about the smoking cessation service and referral pathway, structured/mandatory training, increased physical presence of smoking cessation advisors, communication skills training and paper resources to give patients.

### Focus groups

Twenty-one nurses participated in focus groups (*n* = 5) and interviews (*n* = 2). Nurses worked in secondary care, patient-facing roles, covering three local hospitals. Almost 50% of participants were nurses working within respiratory settings (*n* = 10), while the remaining nurses covered areas such as maternity care (*n* = 1), medicine (*n* = 2), trauma and rehabilitation (*n* = 1), alcohol service (*n* = 2), adult mental health (*n* = 1), care of older people (*n* = 1) and accident and emergency (*n* = 2). Focus groups lasted between 32 and 69 min and had between two and six participants. Two scheduled focus groups only had one participant attend, so were interviews, each lasting 52 min.

Factors influencing nurses discussing smoking cessation and referring patients to smoking cessation services were identified across 10 of the 14 TDF domains, with no data coded to *goals*, *intentions*, *optimism* and *behavioural regulation*. Findings are presented for the remaining domains, in order of relevance to attaining the target behaviours, with the barriers and facilitators highlighted. These include: Role identity, Knowledge, Environmental context and resources, Memory, attention and decision-making, Social influences, Belief about consequences, Reinforcement, Belief about capabilities, Emotion, and Skills. Illustrative quotes are provided to support the identified themes, with the focus group number and nurse role stated.

#### Role identity

All nurses identified discussing smoking and promoting cessation as an important part of their role, which may thus facilitate these behaviours. Capturing smoking status when taking a patient history allowed them to gain a holistic understanding of the patient and plan care accordingly:

*“I think it is key to knowing the story of the patient really because if you’re going to plan a person-centred care… it’s difficult to meet their needs authentically” (FG3- Alcohol Nurse Practitioner).*

Those working in respiratory specialities areas perceived smoking to be more relevant to their role, felt more comfortable having conversations, and did so more frequently, whilst nurses in other specialised roles found the topic more daunting and difficult:

*“Absolutely fine*,* but I think this is because it’s part of our role to do that as well. I’m not saying that it’s not part of everybody’s role*,* because it is*,* but I think being in respiratory …” (FG2-Respiratory Nurse).*

Nurses situated later in the patient care pathway felt that the topic should be raised by nurses and other healthcare professionals (i.e., paramedics) while transported to hospital, however this contrasted with views of admission/A&E nurses, who reported competing pressures, rarely having sufficient time to discuss smoking within their role:

*“At the front door the questions should be asked*,* shouldn’t they? It’s not always filled in*,* but the questions should be asked on clerking.” (FG2-Respiratory Nurse)*

#### Knowledge

All nurses had good knowledge of the risks of smoking and its impact on various medical conditions and were able to tailor this information to the patient’s condition or situation. However, procedural knowledge of the ask, advise and refer process varied, suggesting a potential barrier. When raising the issue of smoking with patients, most nurses articulated the questions they were required to ask the patient, and described how they would raise the conversation. However, many lacked knowledge about available support. Some described the behavioural and psychological support provided by HMQ, but others focused only on the pharmacological component and provision of Nicotine Replacement Therapy (NRT):

*“It’s that regular*,* personal connection as well*,* you have got an advisor supporting you*,* ringing up and saying how are you doing*,* […] having that regular contact is better than just having the nicotine replacement” (FG7- Respiratory Nurse Specialist)*

Nurses also had limited procedural knowledge about the referral process for HMQ. Some were completely unaware of how to refer, whilst others knew of varying referral methods (e.g., verbal referral, sending an email or using the internet page), but were unclear of the correct/preferred route. Such inconsistencies were a barrier to referring patients:

“*Well*,* I guess it’s knowing how to refer because myself*,* I don’t actually know how to refer to the stop smoking*,* if it’s simple*,* I don’t know if it’s on the intranet*,* I wouldn’t know where to start…” (FG3- Alcohol Nurse Practitioner)*

#### Environmental context and resources

##### Setting

Some nurses felt that the busy, chaotic context of hospital inpatient settings was inappropriate for having an authentic conversation with a patient about smoking. They believed that having access to a private, calmer area could facilitate discussions, which was echoed by nurses working in outpatient and clinic settings that were more conducive to sensitive discussions with patients about smoking:

*“I work in a clinic setting*,* in that clinic setting it is quite easy to have that conversation. I often work on the wards as a respiratory nurse*,* [it] is much more difficult if the patient’s on the ward. It’s much busier*,* it’s noisier*,* there’s no place you can take the person to speak to privately” (FG7- Respiratory Nurse Specialist)*

##### Prompts and cues

The majority of nurses identified environmental cues that prompted them to raise the issue of smoking and/or refer patients to HMQ, including patient factors/conditions physical presence of smoking cessation advisors and nursing documentation.

##### Patient conditions

The patient’s health condition often prompted nurses to discuss smoking, particularly if smoking exacerbated and increased medical symptoms, or if treatments were contraindicated for patients who smoked (e.g., oxygen therapy):

*“We tend to only ask our respiratory patients when they it’s a medical need for them to stop smoking there’s nothing else that is indicating or prompting us really to have those conversations with other patients” (FG1- Trauma and Rehabilitation Deputy Manager)*

The use of smoking cessation remedies also facilitated smoking cessation discussions. For example, abstinence from smoking during a hospital admission with support of NRT often provided an opportunity for nurses to discuss with patients ongoing abstinence and quitting after discharge:

*“It’s easier to have the conversation after [they have used NRT]*,* as [we can say] ‘you’ve managed now this far and you know are you sure you don’t want to continue with the replacements rather than start smoking again’” (FG1- Medicine Staff Nurse)*

##### HMQ presence

Physical presence and availability of HMQ advisors in hospital settings facilitated referrals, by continuously emphasising the importance of the topic, increasing familiarity with the service and allowing for in-person referrals:

*“They (HMQ advisors) kind of do a little bit of a trawl around the ward*,* our self-admission wards. So*,* the staff and the patients can speak*,* once there … got them on side a couple of times a week” (FG2-Respiratory Nurse Specialist)*

However, the level of interaction with advisors varied, and nurses who had little or no interaction tended to be more sceptical about the service and availability of advisors. Nurses believed that a closer working relationship with advisors could build trust and increase referrals to the smoking service, emphasising the importance of multi-disciplinary working:

*“We could really do with smoking cessation coming down*,* […] perhaps that’s a step that maybe we’re missing and they don’t come down in the day time to see if we have any patients that are gonna be admitted with a respiratory history of who was still smoking” (FG5- A&E Department Manager)*

##### Documentation

Standard nursing assessment documentation includes questions about patient smoking status. Most nurses reported asking about, and recording, the patient’s smoking status at the point of admission. However, few nurses took the opportunity to follow-up on the answers, meaning patients were identified as smokers, but not advised on the benefits of quitting or referred to HMQ:

*“As part of our nursing pack*,* {smoking} is broached within EDS [Emergency Department] …but it’s just literally a question. Do you smoke? Yes/no kind of […] but it doesn’t go much further than that*,* let’s be honest” (FG1- A&E Ward Manager)*

Inclusion of this question on admission documentation meant that some ward-based nurses assumed that smoking had been addressed by other healthcare professionals, and therefore were not prompted to broach the conversation when admitted to ward care:

*“Yeah. I think like we previously said on the WNCR [Wales Nursing Care Record] is usually done a lot on admission wards. So*,* there’s no prompt then to carry on having those conversations on the wards when they come to us” (FG1- Trauma and Rehabilitation Deputy Manager)*

In contrast, others felt that having this information on admission facilitated subsequent conversations, allowing the nurse to ‘prepare’ to engage with the patient about the topic:

*“A lot of the patients we see when we look at their notes first there is a question within the clerking in*,* so you’ve got a rough idea before you go in*,* if they smoke […] which does make it easier for us*,* we’re not the first one asking” (FG3- Alcohol Nurse Practitioner)*

Therefore, prompts and cues within the environment were not only influential on promoting discussions and referrals but were critical to the nurses’ decision-making processes.

#### Memory, attention & decision-making

##### Prioritisation

Nurses worked in high-pressured roles, with competing demands, and identifying an opportunity to discuss smoking was challenging with acutely unwell patients, thus conveying this as a barrier. Competing priorities meant that nurses needed to make conscious decisions about whether to prioritise smoking cessation with the patient, and how receptive they would be to this conversation:

*“Like I said when they’re coming into us*,* they’re acutely unwell so perhaps their frame of mind isn’t in the place at that time to sort of consider at that moment giving up smoking. It’s not quite straightforward with us” (FG4- Senior Mental Health Nurse)*

Nurses’ decision-making appeared to focus on short term rather than longer term implications. Addressing smoking was often not prioritised as it did not require immediate intervention, and the consequences were minimal compared to other contextual burdens (e.g., withdrawal from alcohol), which were harder for nurses to manage:

*“I suppose again in A&E when we’re asking that*,* it’s the smoking and drinking history so we would become more hyper alert if somebody’s a drinker because obviously that’s gonna cause us issues in A&E much quicker than if somebody’s a smoker” (FG5- A&E Department Manager)*

##### Time and staff capacity

The majority of nurses consistently reported a lack of time to discuss smoking cessation as a barrier. This emphasises the importance on nurses’ decision-making and underlying perceptions about discussing smoking cessation. Prioritisation of tasks is influenced by the nurses’ professional judgement of their situation and the perception that the discussion about smoking may be heavy and extensive. For example, those working in areas with high patient footfall, e.g., A&E, and those delivering specialist clinics, disclosed that they struggled to address all presenting problems within the limited time available:

*“I think time management as well for us in ED is a big thing just because the level and the volume of patients that are coming through the door*,* it’s maybe not a conversation that we can always have” (FG1- A&E Ward Manager)*

This echoes previous domains such as role identity, where nurses’ expectations of themselves and colleagues were unclear, along with the lack of clarity of task expectations to deliver smoking cessation interventions.

#### Social influences

From an organisational perspective, some nurses reported a lack of recognition and support from senior management relating to the challenging, and sometimes hostile, reactions of patients when addressing smoking cessation:

*“It’s about the those up there*,* the health board recognising the challenges*,* how hard it is … it might be very challenging all over the place*,* but we do feel it’s probably more challenging in mental health and we would just like that acknowledged that a job is very difficult when it comes to managing violence and aggression and verbal abuse” (FG4- Senior Mental Health Nurse)*

Nurses experienced, and anticipated, a variety of reactions from patients when discussing smoking. Some described successes where patients agreed to make a quit attempt, whereas others reported being met with aggression, hostility and/or denial from patients. Using cost-benefit analysis [49] of the issue, and thus needing to adjust their approach, increasing psychological burden on them:

*“I don’t think we’ve got any reason not to ask*,* but sometimes obviously being in ED*,* there are aggressive patients who don’t really want a conversation of any kind. So*,* you know you sort of manage your situation around your patient really” (FG1- A&E Ward Manager)*

Some nurses believed that patient’s resistance to discussing smoking and making a quit attempt, resulted from their age, smoking history or perceptions that *“the damage is done” (FG2-Respiratory Nurse Specialist)*:

*“I think because the majority of our patients are really elderly*,* they*,* you know*,* they’ve smoked all their lives*,* they’re not really happy to stop*,* to be honest with you” (FG1- Care of the Elderly*,* Ward Manager)*

Presence of patient family members had mixed effects, with some nurses identifying their ability to motivate patients to engage with HMQ services and support abstinence, whilst others experienced family members reinforcing smoking behaviours (e.g., taking them away from the ward to have a cigarette).

Negative past experiences and anticipated responses seemingly influenced nurses’ beliefs about the impact of intervening, and thus may explain why some nurses do not, or inconsistently, perform the target behaviours.

#### Belief about consequences

Beliefs around the HMQ service and how they could support patients influenced the likelihood of nurses referring. Some nurses felt apprehensive that the service would support patients who they referred, due to limited, or no, feedback from the service about past referrals:

*“I would hope that it would be picked up but because there’s no feedback to know that they’ve engaged*,* to know that there’s been change” (FG3- Alcohol Nurse Practitioner)*

Those who received anecdotal feedback from HMQ tended to be more confident in the service and its provisions.

#### Reinforcement

Nurses suggested that service-level feedback on outcomes would encourage smoking cessation discussions and increase confidence that patients would be supported by HMQ following referral. Feedback would also allow them to promote the service to other patients:

“*Knowing how good a service is*,* that you can help promote the service” (FG1-A&E Ward Manager)*

*“That would be good to see because then the staff can see then and the patients that it does actually work and they do get followed up” (FG5- Respiratory Ward Manager)*

Additionally, acknowledgment of referrals and patient-level feedback from HMQ on engagement and outcomes would foster a sense of continuity of care, motivating and facilitating nurses to continue discussing smoking and making referrals:

*“Maybe if they receive feedback on*,* oh*,* you referred this patient*,* and they stopped smoking*,* […] That would be quite good. And when you get a nice thanks for it*,* it makes you feel worthwhile. So that would probably help motivate nurses. They can use it as feedback for the revalidation as well” (FG6- Maternity care)*

#### Belief about capabilities

Despite confidence in raising the issue, nurses held limiting beliefs about their ability to influence a patient’s decision or motivate them to engage with smoking cessation, acknowledging that it is dependent on patient choice:

*“But if she’s declined*,* she’s declined. You can’t do anything about it” (FG6-Maternity care)*

One nurse stated that patients are “*quite open and honest if they don’t want to give up*,* we just make sure they understand the implications of that” (FG2- Respiratory Staff Nurse).*

Nurses working in mental health and alcohol services experienced difficult conversations more often, but emphasised how constant exposure increased their confidence to discuss smoking cessation:

*“I’ve always worked with addiction*,* so it’s always been quite normal to talk about it*,* but I remember when I was a student*,* I was quite daunted to ask questions like that…” (FG3-Alcohol Nurse Practitioner)*

*“Conversations with our patients about giving up smoking are not easy” (FG4- Senior Mental Health Nurse)*

#### Emotion

Addressing smoking with patients evoked an emotional reaction for some nurses, which could potentially influence and act as a barrier to future discussions:

*“You just feel like always disheartened. You’re like*,* oh*,* well*,* I was trying to help and they didn’t take it that way … you’re always afraid to approach it again because you don’t know what their reaction is going to be” (FG6- Maternity care)*

Nurses also described feelings of frustration from both patients and practitioners following negative interactions, particularly when attempts to address the issue of smoking result in patient disengagement and not resolving the primary health concern:

*“You’ve achieved no progress for either presenting problem. It’s frustrating for the patient*,* it’s frustrating for the practitioner as well” (FG3- Alcohol Nurse Practitioner)*

#### Skills

Nurses felt that there was a certain skill to establishing a meaningful, trusting relationship with a patient to talk about a *“taboo subject*” *(FG6- Maternity care)* such as smoking:

*“We try and establish trust with the patient because obviously that’s key to open disclosure…” (FG3- Alcohol Nurse Practitioner)*

Thus, some nurses felt the need for adequate, dedicated and up-to-date training to fulfil the expectation to have conversations discussing smoking cessation:

*“It’s probably more a lot about training and development because I don’t think as an organisation some of us have not had smoking cessation training for quite a long time” (FG1- A&E Ward Manger)*

## Discussion

This mixed methods study investigated barriers and facilitators to secondary care nurses discussing smoking with patients and referring them to the Welsh smoking cessation service, Help Me Quit (HMQ). Facilitators included nurses seeing these behaviours as consistent with their role and presence of prompts in the environment, whilst barriers included lack of and inconsistent referral methods, competing priorities and patient resistance. Identifying and understanding these barriers and facilitators is important to enable the development of interventions to support nurses to discuss and refer patients to smoking cessation services, ultimately promoting the health of patients in their care.

In line with the mixed methods approach, key findings from the concurrent survey and focus groups are integrated [35] and are discussed in turn: the role of nurses in promoting smoking cessation, discrepancies between “ask” and “refer” behaviours, limited knowledge of smoking cessation service pathway and outcomes, and perceptions of patient and anticipated negative reactions.

### The role of nurses in smoking cessation promotion

Nurses across the survey and focus groups reported feeling comfortable when discussing smoking cessation, due to good knowledge of the risks and impact of smoking and the perception that it was part of their role. Agreement that discussing smoking and referring patients was part of their social/professional role significantly predicted greater self-reported rates of these behaviours from nurses in the survey. A recent scoping review of 53 articles categorised the roles that nurses perform in smoking interventions, including assessors, educators, practice facilitators, coordinating collaborators, organisers and supervisors, concluding that nurses play a vital role [31]. Conversely, free-text comments in a large Danish mixed methods survey revealed that smoking cessation was not perceived by healthcare professionals as part of their role [50].

Nurses in the focus groups in our study who deemed smoking more relevant to their role (e.g., respiratory nurses) routinely integrated smoking discussions within delivery of standard care and treatment interventions. However, high-pressured roles, competing job demands, and patient acute conditions often resulted in cognitive dissonance [51] between beliefs about the importance of discussing as part of the nurse’s role, and beliefs in their ability to raise the conversation in practice. Similarly, nurses in a Dutch qualitative study raised competing care demands and lack of self-efficacy as important barriers to supporting smoking cessation with disadvantaged young women during and following pregnancy [52].

Nurses appeared to anticipate that smoking discussions would be lengthy and difficult, which further deterred them from raising the issue when managing competing priorities. The Danish mixed-methods survey reported that healthcare professionals were concerned about offending patients and were concerned it would impact their rapport [50]. Opinions regarding the most appropriate time, place or healthcare professional to have these discussions also varied and were consequential to nurses’ varied expectations of themselves and colleagues. Some suggested discussions should be undertaken at clerking, whilst those working ‘*at the front door’* highlighted the unrealistic expectation that colleagues hold of them due to the fast-paced environment they work within and felt that ward/clinic staff were better placed to intervene. A review of Cochrane reviews of tobacco cessation interventions concluded that even brief, opportunistic advice from a healthcare worker can promote smoking cessation [30]. Clarity and consensus on the expectations of nurses for intervention (i.e., a very brief conversation to ascertain smoking status, deliver brief advice and refer to smoking cessation services, who provide in-depth intervention) may aid nurses to integrate this into their existing practice, and alter perceptions that conversations are likely to be challenging and time consuming.

### Discrepancies between ‘Ask’ and ‘Refer’ behaviours

The NICE quality statement on tobacco mandates that patients who smoke should receive treatment to stop smoking upon admission [25]. Although all nurses in the current study recognised the importance of identifying patient smoking status, the survey identified a discrepancy between self-reported rates of asking about smoking status and subsequently delivering brief advice and making referrals to the hospital smoking cessation service. Focus group participants explained that they would ask patients about smoking status when prompted by standard assessment documentation but would not follow up on responses with brief advice or a referral. This may be explained by barriers discussed by nurses, including limited time and knowledge about how to make referrals, perceived patient resistance to referrals and limited beliefs that making a referral will ultimately result in patient smoking cessation. Similarly, the Danish survey concluded that lack of time and insufficient knowledge on how to support smoking cessation were key barriers for healthcare professionals [50].

Predictive modelling from the survey analysis provided preliminary evidence that increased frequency of Ask and Refer behaviours were predicted by behavioural regulation and social/professional role, whilst memory, attention and decision-making predicted increases in Ask, not Refer, and reinforcement only predicted increases in Refer. Further exploration of these domains as predictors of behaviour is warranted using recorded, rather than self-reported, rates of behaviours and larger samples. This will generate further nuanced understanding of predictors of the individual behaviours and may explain discrepancies between rates of each behaviour.

### Limited knowledge of the smoking cessation service, referral pathways and outcomes

Similar to an earlier Danish study [50], nurses in the current study reported in the free-text survey responses and focus groups that they had limited knowledge of the local smoking cessation service offering and how to make referrals. They perceived referring to the service to be difficult and struggled to explain available support to patients. HMQ offer a breadth of referral methods to increase accessibility, but at times, this resulted in confusion amongst nurses about the best way to refer, particularly where there were inconsistencies between hospital sites, settings and speciality areas. Nurses in the current study who had regular contact with the smoking cessation service and were more familiar with specific advisors were more likely to make referrals to the service. Indeed, effective referral systems require collaboration between professionals to facilitate good referral practices [53]. Developing closer working relationships between nurses and smoking cessation advisors and increased physical presence of advisors in their environment may serve as cues for behaviours and increase familiarity with the service.

Furthermore, nurses received limited feedback on the outcomes of referrals at either service or patient level, which influenced their trust in the service and beliefs that making a referral would be beneficial for patients. A scoping review characterised features of effective referral systems and identified a feedback process as one important feature [53]. Receiving reinforcement, for instance through seeing examples of where behaviours have resulted in successful outcomes for patients, was predictive of increased self-reported rates of making referrals in the current study. Further exploration of the impact of reinforcing nurse behaviour through delivering feedback would be beneficial.

### Perceptions about patients and anticipated negative reactions

Healthcare professionals’ perceived lack of patient motivation as a key barrier to smoking cessation support in a secondary care mixed methods study in Denmark [50]. In our study, nurses in the survey and focus groups often felt that they were unable to influence a patients’ decision to engage with the service and make a quit attempt. At times, this was influenced by preconceived opinions about the patient’s age, smoking history or medical condition. Cognitive and implicit biases and the detrimental impact on nurses’ judgment and decision-making, and ultimately patient outcomes, were highlighted as concerns in a recent scoping review of 77 sources [54]. The review also reported the dearth of intervention studies to reduce these biases. In the current study, past experiences of aggression, hostility, patient denial and negative reactions to conversations also influenced their willingness to discuss and promote smoking cessation. Nurses had the additional cognitive load of assessing the risks and benefits of engaging the patient in conversation.

This was especially challenging in nurse specialities that were more likely to experience patients with challenging behaviours, such as mental health and A&E. Nurses working in such environments may benefit from specialised communication skills training to enable them to adapt interventions and conversations to meet the needs of their patients [55]. Additional training around communication skills was identified as a need for some survey respondents, and receiving training relating to smoking was associated with more positive responses on eight of the TDF domains, including knowledge, skills and beliefs about capabilities. A recent systematic review examined the effect of smoking cessation training on student nurses’ learning outcomes, and reported enhancements to students’ attitudes/motivation towards smoking cessation, self-efficacy and practice of the 5 A (Ask, Advise, Assess, Assist, Arrange) approach [56]. Similarly, a one-day smoking cessation training programme undertaken by nurses in Japan contributed to an increase in the frequency of nurses providing smoking cessation advice in line with the 5 A approach after three months, although the response rate was 46% [57].

Training on psychologically informed communication skills may help nurses to build trusting relationships with their patients, manage their complex needs and feel more comfortable to have difficult conversations about health behaviour change. For example, a technique such as Motivational Interviewing has demonstrated significant positive impact on reducing smoking behaviour in patients when adopted into practice [58]. Of note, a recent scoping review identified that Motivational Interviewing and the 5 A method are the most used techniques for smoking cessation by nurses in published interventional studies [31]. Additionally further research has demonstrated delivering public health interventions such as Making Every Contact Count [26] are also effective in delivering brief behaviour change interventions. However, uptake of training tends to be low and nurses can experience challenges implementing new skills in practice [59, 60].

### Practical implications

The findings from the present study will inform phase 2 of the Think Quit study [61], using the Behaviour Change Wheel [34] to co-develop an intervention with nurses and stakeholders to address identified barriers and determinants of the target behaviours. The research team have generated a series of recommendations for practice that correspond with identified barriers (Table 4). Phase 2 will involve prioritising barriers using the APEASE (Acceptability, Practicability, Effectiveness, Affordability, Side-effects, Equity) criteria [34] to determine which recommendation to pursue within the constraints of the Think Quit study.

**Table 4:** Recommendations for practice corresponding with identified barriers, using the BCW

Intervention Function	TDF Domains Targeted by the Intervention	Barrier Identified	Recommendations
Education	Knowledge Professional Role and Identity Belief about Capabilities	Lack of knowledge of the referral pathway to HMQ Limited knowledge of HMQ service provisions Lack of role clarity and expectations	Provide guidance on the referral pathway Provide information/ resources on the HMQ service offer Provide guidance on the role of nurses and clarity around expectations
Incentivisation	Goals Reinforcement Emotion	Lack of feedback from HMQ	Provide feedback on service and/or patient-level outcomes following referrals (e.g., number of referrals that engaged in the service and numbers of those who engaged who successfully quit)
Training	Skills Environmental Context and Resources	Lack of skills to deliver difficult conversations	Deliver person-centred care and communication skills training (i.e., MI), in a format/setting that is conducive to the demands on nurse time and the context they work within
Environmental restructuring	Memory, Attention and Decision Processes Reinforcement Environmental Context and Resources Social Influences	Insufficient prompts and resources Complexity in the referral pathway Lack of HMQ advisors’ presence	Include additional prompts and environmental cues for behaviours in nursing documentation and settings Make the referral pathway to smoking cessation services simple and seamless Increase HMQ advisors’ presence in hospital settings
Modelling	Professional Role and Identity Belief about Consequences Social Influences	Lack of clarity on what good discussions about smoking cessation involves Lack of peer support from colleagues and senior leaders	Provide examples of smoking cessation discussions and methods to refer Offer opportunities for staff to shadow conversations or view case studies of successful referrals
Enablement	Memory, Attention and Decision Processes Environmental Context and Resources Social Influences	Lack of HMQ advisors’ presence Staff shortages, capacity and time	Increase managerial and organisational support and recognition of challenges experienced by nurses Strengthen the relationship and collaborative working between the smoking cessation service and nurses Include nurse champion roles amongst nursing staff

### Strengths

The mixed methods design allowed for rich in-depth insights from focus group data to be integrated with quantitative and qualitative survey responses from a larger sample of nurses with varied specialities and experiences. Systematic application of behavioural science theories enabled the researchers to identify determinants of the two target behaviours, which will subsequently inform intervention co-development in Phase 2. The study benefited from collaboration between health psychology, nursing and public health specialists when interpreting the findings and developing recommendations for practice.

### Limitations

While the study provides insight through an evidence-based framework, the deductive nature of the coding may have resulted in themes relating to other relevant behaviours, actors (e.g., patients, other healthcare professionals) or factors outside of the TDF being missed [40]. At times focus group data were coded to multiple TDF domains, but domains were analysed and reported separately. To mitigate this, findings have been integrated across domains in the discussion, highlighting connections between domains and supplemented by findings from the survey.

The study faced significant challenges recruiting nurses and despite extensive and varied recruitment efforts, recruitment targets were not met. We therefore recognise that the perspectives reported here may not represent those of the broader nursing community within the organisation. The majority of focus group participants worked in respiratory settings, so findings may be more relevant to specialities closely aligned with smoking. A breadth of specialities was represented in the survey responses; however, sub-samples were too small to undertake sub-group analysis between specialities. Finally, the survey did not used a validated tool for the TDF questions and was reliant on self-reported frequency of behaviours, limiting confidence in predictive modelling between TDF domains and behaviours.

### Implications for research

The research team are using these findings to co-develop an intervention that empowers nurses to discuss smoking cessation and refer to specialist support. Several nurses in this study believed that addressing smoking cessation is the responsibility of all healthcare professionals. Future research should investigate perceptions and experiences of the two target behaviours among a broader sample of healthcare professionals. Data were analysed deductively using the TDF and future analyses could benefit from adopting a combination of deductive and inductive coding methods to allow a wider exploration beyond the theoretical framework. Recruitment challenges signify the need to ensure that the research methodology and methods align with the needs, preferences and context of nurses to increase engagement, facilitate the development of evidence-based interventions, and demonstrate best practice in nursing research.

## Conclusions

Nurses in this study recognised the importance of their role in discussing smoking but lacked knowledge about smoking cessation services and experienced contextual barriers limiting their ability to refer patients to services. Reducing barriers to discussing smoking and referring patients could help nurses to support their patients to achieve the best health outcomes by quitting smoking. Interventions to address identified barriers should account for competing priorities and time pressures experienced by nurses. Co-development with nurses will increase the likelihood that interventions are feasible, acceptable and impactful across a range of settings within which nurses work. The next step is to select appropriate barriers(s) from phase 1 and work with nurses to co-develop a behaviourally informed intervention to address these.

## Electronic supplementary material

Below is the link to the electronic supplementary material.

Supplementary Material 1: GRAMMS: This supplementary document outlines the application of the GRAMMS guidelines that were adhered to when preparing the manuscript.

Supplementary Material 2: Data collection tools: This supplementary document includes the data collection tools used to inform and gather data in the Think Quit Study. Example Survey: Pages 2–6. Focus Group Guide: Pages 7–9

Supplementary Material 3: Supplementary survey analysis: This supplementary document includes further detail of all statistical analysis undertaken at sub-group level, which is presented in summary format in the main manuscript.

## Data Availability

The datasets used and analysed during the current study are available from the corresponding author on reasonable request. The survey and focus group schedule are supplied in Additional File 1.

## Abbreviations

A&E: Accident & Emergency
APEASE: Acceptability, Practicability, Effectiveness, Affordability, Side-effects, Equity
BCW: Behaviour Change Wheel
COM-B: Capability, Opportunity, Motivation - Model of Behaviour
COPD: Chronic Obstructive Pulmonary Disease
EDS: Emergency Department
FG: Focus group
HMQ: Help Me Quit
NICE: National Insitute for Health and Care Excellence
NRT: Nicotine Replacement Therapy
TDF: Theoretical Domains Framework
WNCR: Wales Nursing Care Record
