# Supplementary material for: A mixed methods study exploring barriers and facilitators to secondary-care nurses discussing smoking cessation with patients: phase 1 of the Think Quit Study
Source: BMC Nurs. 2025 Aug 5;24:1020. doi: 10.1186/s12912-025-03597-6 (PMC12323258; doi:10.1186/s12912-025-03597-6)
Supplement: Supplementary file 1 — Supplementary Material 1: GRAMMS: This supplementary document outlines the application of the GRAMMS guidelines that were adhered to when preparing the manuscript. [file 12912_2025_3597_MOESM1_ESM.docx]

**ADDITIONAL FILE 1: Good Reporting of A Mixed Methods Study (GRAMMS) guideline**

| **Guideline** | **Application** | **Section** |
| --- | --- | --- |
| Describe the justification for using a mixed methods approach to the research question | The convergent mixed-methods design is justified. | Methods: Design and setting |
| Describe the design in terms of the purpose, priority and sequence of methods | The purpose and sequencing of the parallel approach is described. | Methods: Design and setting, Data Collection |
| Describe each method in terms of sampling, data collection and analysis | Each method is outlined, including sampling, data collection and analysis | Methods: Participants, Data collection |
| Describe where integration has occurred, how it has occurred and who has participated in it | The approach to data integration is outlined | Methods: Design and setting |
| Describe any limitation of one method associated with the presence of the other method | The study limitations are acknowledged | Discussion: Limitations |
| Describe any insights gained from mixing or integrating methods | The study strengths are outlined | Discussion: Strengths |

O'Cathain A, Murphy E, Nicholl J. The quality of mixed methods studies in health services research. J Health Serv Res Policy. 2008;13: 92-98.
