# Supplementary material for: A mixed methods study exploring barriers and facilitators to secondary-care nurses discussing smoking cessation with patients: phase 1 of the Think Quit Study
Source: BMC Nurs. 2025 Aug 5;24:1020. doi: 10.1186/s12912-025-03597-6 (PMC12323258; doi:10.1186/s12912-025-03597-6)
Supplement: Supplementary file 2 — Supplementary Material 2: Data collection tools: This supplementary document includes the data collection tools used to inform and gather data in the Think Quit Study. Example Survey: Pages 2–6. Focus Group Guide: Pages 7–9 [file 12912_2025_3597_MOESM2_ESM.docx]

**ADDITIONAL FILE 2: DATA COLLECTION TOOLS**

This supplementary document includes the data collection tools used to inform and gather data in the Think Quit Study.

Both documents were informed using the Theoretical Domains Framework:

1. Example Survey: Pages 2-6
2. Focus Group Guide: Pages 7-9

**ABOUT YOUR ROLE**

What is your current job title?

|  |
| --- |

What speciality area of nursing do you work in? (Please circle)

| Surgery | Medicine | Acute medicine | Intensive care | Emergency medicine |
| --- | --- | --- | --- | --- |
| Respiratory | Cardiology | Care of the elderly | Trauma & Orthopaedics | Outpatients |
| Maternity & midwifery |  |  |  | Other (please specify): ___ |

How long have you been a nurse? (Please circle)

| Less than 1 year | 1-2 years | 3-5 years | 6-10 years | Over 10 years |
| --- | --- | --- | --- | --- |

What Agenda for Change band are you currently working at? (Please circle)

| Band 5 | Band 6 | Band 7 | Band 8a-d |
| --- | --- | --- | --- |

Is your current role patient-facing? (Please circle)

| Yes | No |
| --- | --- |

Would you consider the area you work in to have a high turnover of patients? (Please circle)

| I see new patients everyday | I see new patients every week | I see the same patients on a regular basis | I look after the same patients for a long period of time |
| --- | --- | --- | --- |

Which CTM UHB hospital do you work in? (Please circle)

| Prince Charles Hospital (PCH) | Princess of Wales Hospital (POW) | Royal Glamorgan Hospital (RGH) | Ysbyty Cwm Cynon (YCC) |
| --- | --- | --- | --- |
| Ysbyty Cwm Rhondda (YCR) | Cefn Yr Afon | Dewi Sant Health Park | Glanrhyd Hospital |
| Keir Hardie University Health Park | Maesteg Community Hospital | Merthyr Renal Dialysis Unit | Pontypridd Cottage Hospital |
| Ysbyty George Thomas | Pinewood Hospital | I work across multiple hospital sites | Other (please specify):  _________________ |

ABOUT YOU

What is your ethnicity? (Please circle)

| White (includes Welsh, English, Scottish, Northern Irish, British, Gypsy or Irish Traveller, any other White Background | Mixed or multiple ethnic groups (includes White and Black Caribbean, White and Black African, White and Asian, any other Mixed or Multiple ethnic background) | Asian or Asian British (includes Indian, Pakistani, Bangladeshi, Chinese, any other Asian background) |
| --- | --- | --- |
| Black, African, Caribbean or Black British (includes any other Black, African or Caribbean background) | Prefer not to disclose my ethnic group | Other ethnic group (Arab or any other ethnic group, please specify): _____________________ |

What is your smoking status? (Please circle)

| Current smoker | Current e-cigarette/ vape user | Ex-smoker | Never smoked |
| --- | --- | --- | --- |

What training have you previously undertaken in relation to discussing smoking and smoking cessation? *(Circle all that apply)*

| NHS ESR training | Making Every Contact Count (MECC) Level 1 | Making Every Contact Count (MECC) Level 2 | Briefing/training from Help Me Quit team |
| --- | --- | --- | --- |
| Brief Advice Training | National Centre for Smoking Cessation and Training (NCSCT) | Motivational Interviewing training | Other (please specify): ___ |

In your current role, how often do you….

|  | Never | Rarely | Sometimes | Often | Always |
| --- | --- | --- | --- | --- | --- |
| Ask patients about their smoking status? |  |  |  |  |  |
| Record smoking status on the Welsh Nursing Care Record (WNCR)? |  |  |  |  |  |
| Inform patients that they cannot smoke or vape on hospital grounds, as per the CTM UHB Smoke Free Policy? |  |  |  |  |  |
| Provide very brief advice and messages regarding quitting smoking? |  |  |  |  |  |
| Refer patients who are smokers to Help Me Quit? |  |  |  |  |  |
| Support your patient to stay smoke free and be comfortable during their stay by offering and prescribing Nicotine Replacement Therapy (NRT)? |  |  |  |  |  |

In your current role, how important do you think it is to ask patients about their smoking status and refer them to Help Me Quit?

| Not important at all | Slightly important | Important | Very important | Absolutely essential |
| --- | --- | --- | --- | --- |

**TDF**

When thinking about **discussing smoking with hospital patients and referring them to Help Me Quit (HMQ)**, please read the following statements and choose which answer applies to you.

*N.B. HMQ in Hospital stands for Help Me Quit in Hospital.*

|  |  | Strongly disagree | Disagree | Neither agree nor disagree | Agree | Strongly agree |
| --- | --- | --- | --- | --- | --- | --- |
| 1 | I have good KNOWLEDGE of the risks of smoking and the benefits of quitting |  |  |  |  |  |
| 2 | I have good KNOWLEDGE of the Help Me Quit in Hospital service |  |  |  |  |  |
| 3 | I have good KNOWLEDGE of how to refer patients to Help Me Quit in Hospital |  |  |  |  |  |
| 4 | I have good KNOWLEDGE of the CTM UHB Smoke Free Hospital Policy |  |  |  |  |  |
| 5 | I have good KNOWLEDGE of the importance of offering and prescribing NRT for smokers during their hospital stay |  |  |  |  |  |
| 6 | I know WHY it is important to talk to patients about smoking and refer them to HMQ in Hospital |  |  |  |  |  |
| 7 | I know HOW to have sensitive discussions with patients about smoking and refer them to HMQ in Hospital |  |  |  |  |  |
| 8 | I have had sufficient TRAINING in discussing smoking and referring to HMQ in Hospital |  |  |  |  |  |
| 9 | I have had the opportunity to PRACTICE discussing smoking and referring patients to HMQ in Hospital regularly |  |  |  |  |  |
| 10 | Referring patients to HMQ in Hospital is too COMPLEX and difficult |  |  |  |  |  |
| 11 | Discussing smoking with patients is part of my JOB ROLE |  |  |  |  |  |
| 12 | Referring patients to HMQ in Hospital is part of my JOB ROLE |  |  |  |  |  |
| 13 | I feel CONFIDENT in discussing smoking referring patients to HMQ in Hospital |  |  |  |  |  |
| 14 | I do not know where to start and what the STEPS would be in terms of discussing smoking and referring patients to HMQ in Hospital |  |  |  |  |  |
| 15 | I believe I have the skills to discuss smoking and refer patients to HMQ in Hospital |  |  |  |  |  |
| 16 | I believe discussing smoking and promoting smoking cessation is/would achieve the BEST OUTCOME for the patients I care for |  |  |  |  |  |
| 17 | I recognise the VALUE of discussing smoking and referring patients to HMQ in Hospital |  |  |  |  |  |
| 18 | I am CONFIDENT that patients will engage with HMQ in Hospital if I refer them |  |  |  |  |  |
|  |  | Strongly disagree | Disagree | Neither agree nor disagree | Agree | Strongly agree |
| 19 | I am CONFIDENT that the HMQ in Hospital service will support patients in a sensitive and timely manner |  |  |  |  |  |
| 20 | I have seen lots of EXAMPLES where discussing smoking and referring patients to HMQ in Hospital has resulted in successful outcomes for patients |  |  |  |  |  |
| 21 | I INTEND to discuss smoking and refer patients to HMQ in Hospital in the future |  |  |  |  |  |
| 22 | I PRIORITISE discussing smoking and referring patients to HMQ in Hospital in my role |  |  |  |  |  |
| 23 | I am able to OVERCOME challenges that might impact by ability to discuss smoking and refer patients to HMQ in Hospital |  |  |  |  |  |
| 24 | I am able to manage COMPETING DEMANDS so that I can discuss smoking and refer patients to HMQ in Hospital |  |  |  |  |  |
| 25 | Discussing smoking and referring patients to HMQ in Hospital is a REGULAR part of my practice |  |  |  |  |  |
| 26 | I have enough TIME to talk to patients about smoking and refer them to HMQ in Hospital |  |  |  |  |  |
| 27 | I have access to RESOURCES needed to help me discuss smoking and refer patients to HMQ in Hospital (e.g., guidance, frameworks) |  |  |  |  |  |
| 28 | There are PROMPTS in place to remind me to discuss smoking and refer patients to HMQ in Hospital |  |  |  |  |  |
| 29 | I work with OTHERS who regularly discuss smoking and refer patients to HMQ in Hospital |  |  |  |  |  |
| 30 | I have sufficient SOCIAL SUPPORT from colleagues and leaders to discuss smoking and refer patients to HMQ in Hospital |  |  |  |  |  |
| 31 | I need more leadership SUPPORT to discuss smoking and refer patients to HMQ in Hospital |  |  |  |  |  |
| 32 | I am WORRIED about upsetting patients by discussing smoking and referring them to HMQ in Hospital |  |  |  |  |  |
| 33 | I make a CONSCIOUS DECISION to discuss smoking and refer patients to HMQ in Hospital |  |  |  |  |  |
| 34 | I have ways of MONITORING and RECORDING discussions about smoking and referrals to HMQ in Hospital |  |  |  |  |  |

What do you think are the TOP 5 BARRIERS (if any) to **discussing smoking with hospital patients and referring them to Help Me Quit (HMQ)**? (Please list the most significant barrier first). N.B. If you cannot think of 5, please list as many as you can.

| 1 |
| --- |
| 2 |
| 3 |
| 4 |
| 5 |

Can you suggest **5 IDEAS OR STRATEGIES** that could be used to overcome these barriers? (Please list the most useful or impactful idea first). *N.B. If you cannot think of 5, please list as many as you can.*

| 1 |
| --- |
| 2 |
| 3 |
| 4 |
| 5 |

What else would support you to routinely **discuss smoking with hospital patients and refer them to Help Me Quit (HMQ)** in your role?

|  |
| --- |

Is there anything else you would like to add?

|  |
| --- |

**Focus group topic guide – secondary care nurses**

| Introduction | *Welcome and introduce facilitator. Confirm session recording and participants can withdraw or ask for recording to pause. Emphasize that the transcripts will be anonymised once checked for accuracy.*  *Explain the aim: We want to understand your experience of initiating conversations about smoking and discussing referral to specialist stop smoking support and factors that help/hinder this with* ***HOSPITAL IN-PATIENTS****.*  *No right or wrong answers – interested in your views and experiences.*  *Check if any questions or concerns.* |
| --- | --- |
| Exploratory questions | *Go around room and ask for nursing specialty and role for the recording and context.* |
| Discussions about smoking | - Can you tell us a bit about your experience of initiating conversations about smoking with hospital in-patients? - How do you raise the issue of smoking with patients? - What influences when/how you start a conversation about smoking? - Do you use CO monitors within your practice? If so, how, and what role does this play in your conversations? |
| Recording smoking status | - How do you record smoking status? Is this routinely done? - What influences whether, and how, you record smoking status for a patient? |
| Referring to HMQ in Hospital | - How do you introduce the topic of specialist support? - What do you tell patients about specialist support? - How do you explain the process of referring to specialist support? - What can prevent you from discussing referrals to specialist support with hospital patients? - What might support you in having conversations about referrals to specialist support? |
| TDF | *Refer to the TDF framework prompt questions if specific areas have not been discussed or explored within the focus group to generate further specific insight.* |
| Closing questions | - What else might support you in having conversations about referrals to specialist stop smoking support? - Do you think there are things that you could do/could be done to maximise uptake of service? - Anything else you would like to add about your experiences? |
| Close | *Thank the group for their engagement and inform them of next steps and how we will share the findings.* |

| **Domain (definition)** | **Constructs** | **Prompts** |
| --- | --- | --- |
| 1. Knowledge (An awareness of the existence of something) | Knowledge (including knowledge of condition/scientific rationale), Procedural knowledge, Knowledge of task environment | What skills and knowledge do you draw on when raising/discussing smoking with patients?  What additional skills/knowledge might be helpful?  What training have you had/would you like to have? |
| 2. Skills (An ability or proficiency acquired through practice) | Skills, Skills development, Competence, Ability, Interpersonal skills, Practice, Skill assessment |  |
| 3. Social/professional role and identity (A coherent set of behaviours and displayed personal qualities of an individual in a social or work setting) | Professional identity, Professional role, Social identity Identity, Professional boundaries, Professional confidence, Group identity, Leadership, Organisational commitment | How important do you feel it is to discuss smoking with patients?  Who do you think is best placed to raise the issue of smoking with patients?  What role do you think nurses should play? |
| 4. Beliefs about capabilities (Acceptance of the truth, reality or validity about an ability, talent or facility that a person can put to constructive use) | Self-confidence, Perceived competence, Self-efficacy Perceived behavioural control, Beliefs, Self-esteem Empowerment, Professional confidence | How confident do you feel in raising/discussing smoking with patients? |
| 5. Optimism (The confidence that things will happen for the best or that desired goals will be attained) | Optimism, Pessimism, Unrealistic optimism, Identity | How optimistic are you that patients will engage in stop smoking support?  How confident are you that they will stop smoking on gaining support? |
| 6. Beliefs about Consequences (Acceptance of the truth, reality, or validity about outcomes of a behaviour in a given situation) | Beliefs, Outcome expectancies, Characteristics of outcome expectancies, Anticipated regret, Consequents | How confident do you feel that the service you are referring to will be able to help the patient?    What are the benefits and risks of initiating conversations about smoking/making a referral to specialist support? |
| 7. Reinforcement (Increasing the probability of a response by arranging a dependent relationship, or contingency, between the response and a given stimulus) | Rewards (proximal/distal, valued/not valued, probable/improbable), Incentives, Punishment Consequents, Reinforcement, Contingencies, Sanctions | What encourages you to discuss smoking with patients? |
| 8. Intentions (A conscious decision to perform a behaviour or a resolve to act in a certain way) | Stability of intentions, Stages of change model,  Transtheoretical model and stages of change | How strong is your intention to discuss smoking with patients? |
| 9. Goals (Mental representations of outcomes or end states that an individual wants to achieve) | Goals (distal/proximal), Goal priority, Goal/target setting Goals (autonomous/controlled), Action planning, Implementation intention | How do you plan such conversations?  How often does it go to plan? What gets in the way? |
| 10. Memory, attention and decision processes (The ability to retain information, focus selectively on aspects of the environment and choose between two or more alternatives) | Memory, Attention, Attention control, Decision making, Cognitive overload/tiredness | Is discussing smoking something you usually do?  How do you ensure it is discussed? |
| 11. Environmental context and resources (Any circumstance of a person’s situation or environment that discourages or encourages the development of skills and abilities, independence, social competence and adaptive behaviour) | Environmental stressors, Resources/material resources, Organisational culture/climate, Salient events/critical incidents, Person × environment interaction, Barriers and facilitators | What external factors impact on your ability to raise the issue/discuss referral to specialist support?  What support do you receive/would you like to receive? |
| 12. Social influences (Those interpersonal processes that can cause individuals to change their thoughts, feelings, or behaviours) | Social pressure, Social norms, Group conformity, Social comparisons, Group norms, Social support, Power Intergroup conflict, Alienation, Group identity, Modelling | How do other nurses approach conversations about smoking?  Do you talk to other nurses about approaching conversations about smoking? If not, why not? If yes, what do you talk about? |
| 13. Emotion (A complex reaction pattern, involving experiential, behavioural, and physiological elements, by which the individual attempts to deal with a personally significant matter or event) | Fear, Anxiety, Affect, Stress, Depression, Positive/negative affect, Burn-out | How do you feel about raising the issue of smoking with patients? |
| 14. Behavioural regulation (Anything aimed at managing or changing objectively observed or measured actions) | Self-monitoring, Breaking habit, Action planning | When meeting with patients, how aware are you of the topic of smoking? |
