# Supplementary material for: A mixed methods study exploring barriers and facilitators to secondary-care nurses discussing smoking cessation with patients: phase 1 of the Think Quit Study
Source: BMC Nurs. 2025 Aug 5;24:1020. doi: 10.1186/s12912-025-03597-6 (PMC12323258; doi:10.1186/s12912-025-03597-6)
Supplement: Supplementary file 3 — Supplementary Material 3: Supplementary survey analysis: This supplementary document includes further detail of all statistical analysis undertaken at sub-group level, which is presented in summary format in the main manuscript. [file 12912_2025_3597_MOESM3_ESM.docx]

**ADDITIONAL FILE 3: SUPPLEMENTARY SURVEY ANALYSIS**

**VARIABLES**

***Sub-group: Years of experience***

| Variable | Under 10 years | Over 10 years |
| --- | --- | --- |
| Definition | Self-reported to have under 10 years’ experience as a registered nurse | Self-reported to have over 10 years’ experience as a registered nurse |
| N | 38 | 72 |

***Sub-group: Training experience***

| Variable | No training | Training experience |
| --- | --- | --- |
| Definition | Self-reported to not have received any formal or informal training relating to smoking cessation promotion. | Self-reported to have received one or more form of formal or informal training relating to smoking cessation promotion, including; NHS ESR training, Making Every Contact Count (MECC) training Level 1 and/or 2, Briefing/training from HMQ team, Brief advice training, National Centre for Smoking Cessation and Training, Motivational Interviewing training or other form of training. |
| N | 31 | 79 |

***Sub-group: Smoking status***

| Variable | Never smoked | Smoking history |
| --- | --- | --- |
| Definition | Self-reported to have never smoked. | Self-reported to be either a current smoker, current e-cigarette/vape user or an ex-smoker. |
| N | 78 | 32 |

***Sub-group: Pay Band***

| Variable | Band 5 | Band 6 | Band 7 | Band 8 |
| --- | --- | --- | --- | --- |
| Definition | Self-reported to be working at Agenda for Change Pay Band 5 | Self-reported to be working at Agenda for Change Pay Band 6 | Self-reported to be working at Agenda for Change Pay Band 7 | Self-reported to be working at Agenda for Change Pay Band 8a-d |
| N | 39 | 24 | 37 | 10 |

***Perceived importance***

| **Variable** | **Definition** | **Response range** |
| --- | --- | --- |
| Perceived importance | In your current role, how important do you think it is to ask patients about their smoking status and refer them to Help Me Quit? | Not important at all = 1  Slightly important = 2  Important = 3  Very important = 4  Absolutely essential = 5 |

***Self-reported frequency of behaviours***

| **Variable** | **Definition** | **Response range** |
| --- | --- | --- |
| Ask | Ask patients about their smoking status | Never = 1  Rarely = 2  Sometimes = 3  Often = 4  Always = 5 |
| Record | Record smoking status on the Welsh Nursing Care Record (WNCR) |  |
| Inform | Inform patients that they cannot smoke or vape on hospital grounds, as per the CTM UHB Smoke Free Policy |  |
| Brief advice | Provide very brief advice and messages regarding quitting smoking |  |
| Refer | Refer patients who are smokers to Help Me Quit |  |
| NRT | Support your patients to stay smoke free and be comfortable during their stay by offering and prescribing Nicotine Replacement Therapy (NRT) |  |

***TDF domains***

| **Variable** | **Definition** | **Response range** |
| --- | --- | --- |
| Knowledge | Knowledge | Strongly disagree = 1  Disagree = 2  Neither agree nor disagree = 3  Agree = 4  Strongly agree = 5 |
| Skills | Physical skills |  |
| Soc/prof role | Professional/social role and identity |  |
| Beliefs cap | Beliefs about capabilities |  |
| Optimism | Optimism |  |
| Beliefs cons | Beliefs about consequences |  |
| Reinforcement | Reinforcement |  |
| Intentions | Intentions |  |
| Goals | Goals |  |
| Memory | Memory, attention and decision processes |  |
| Environment | Environmental context and resources |  |
| Social influences | Social influences |  |
| Emotion | Emotion |  |
| Beh regulation | Behavioural regulation |  |

**RELATIONSHIPS BETWEEN RESPONDENT CHARACTERISTICS**

Chi-Square Tests of Independence were used to evaluate whether there was a significant association between categories of variables between four participant characteristics.

Significant relationship between:

- Years of experience and pay band (χ2 (3, 110) = 14.16, p=0.003).

No significant relationship between:

- Pay band and smoking status: χ2 (3, 110) = 1.519, p = 0.6778
- Years of experience and hospital location: χ2 (3, 110) = 1.242, p = 0.743
- Years of experience and smoking status: χ2 (1, 110) = 1.819, p = 0.178
- Years of experience and training experience: χ2 (1, 110) = 0.0168, p = 0.897
- Pay band and hospital location: χ2 (9, 110) = 15.969, p = 0.0675
- Pay band and training experience: χ2 (3, 110) = 1.617, p = 0.656
- Hospital location and smoking status: χ2 (3, 110) = 2.8, p = 0.424
- Hospital location and training experience: χ2 (3, 110) = 1.688, p = 0.64
- Smoking status and training experience: χ2 (1, 110) = 3.452, p = 0.063

**SUB-GROUP ANALYSES FOR PERCEIVED IMPORTANCE, SELF-REPORTED FREQUENCY OF BEHAVIOURS AND TDF DOMAINS REPORTED BY PARTICIPANT CHARACTERISTIC**

1. **Years of experience**

*Perceived importance*

| **IMPORTANCE RATING** | **Under 10 years mean** | **Over 10 years mean** | **Analysis** | **Significant** |
| --- | --- | --- | --- | --- |
|  | 4.0541 | 4.1528 | F(1,107)=0.2569, P=0.6133 | No |

*Behaviours*

| **Behaviour** | **Under 10 years mean** | **Over 10 years mean** | **Analysis** | **Significant** |
| --- | --- | --- | --- | --- |
| Ask | 3.8421 | 4.1111 | F(1,108)=1.2137, p = 0.2731 | No |
| Record | 3.5263 | 2.6250 | F(1,108)=7.0316, p = 0.0092 | Yes |
| Inform | 4.1053 | 3.2500 | F(1,108)=10.2833, p = 0.0018 | Yes |
| Brief advice | 3.5263 | 3.5833 | F(1,108)=0.0529, p = 0.8185 | No |
| Refer | 3.3158 | 3.1806 | F(1,108)=0.2574, p = 0.613 | No |
| NRT | 3.7105 | 3.1111 | F(1,108)=4.4086, p = 0.0381 | Yes |

*TDF Domains*

| **Domain** | **Under 10 years mean** | **Over 10 years mean** | **Analysis** | **Significant** |
| --- | --- | --- | --- | --- |
| Knowledge | 3.8947 | 3.9167 | F(1,108)=0.0273, p = 0.869 | No |
| Skills | 3.0263 | 3.0417 | F(1,108)=0.022, p = 0.8823 | No |
| Soc/prof role | 4.0132 | 3.9722 | F(1,108)=0.0491, p = 0.8251 | No |
| Beliefs cap | 3.3553 | 3.3715 | F(1,108)=0.0313, p = 0.8599 | No |
| Optimism | 4.1316 | 4.2639 | F(1,108)=0.731, p = 0.3944 | No |
| Beliefs cons | 3.7719 | 3.7222 | F(1,108)=0.1515, p = 0.6978 | No |
| Reinforcement | 3.3684 | 3.2639 | F(1,108)=0.2855, p = 0.5942 | No |
| Intentions | 4.0526 | 4.0694 | F(1,108)=0.0099, p = 0.9208 | No |
| Goals | 3.2632 | 3.4722 | F(1,108)=1.6125, p = 0.2069 | No |
| Memory | 3.4211 | 3.3889 | F(1,108)= 0.0282, p = 0.8668 | No |
| Environment | 3.2018 | 3.3194 | F(1,108)=0.4372, p = 0.5099 | No |
| Social influences | 3.2368 | 3.1701 | F(1,108)=0.4934, p = 0.4839 | No |
| Emotion | 3.5000 | 3.7361 | F(1,108)=1.5053, p = 0.2225 | No |
| Beh regulation | 3.5000 | 3.4583 | F(1,108)=0.0534, p = 0.8177 | No |

1. **Smoking status**

*Perceived importance*

| **IMPORTANCE RATING** | **Never smoked mean** | **Smoking history mean** | **Analysis** | **Significant** |
| --- | --- | --- | --- | --- |
|  | 4.1250 | 4.1169 | F(1,107)=0.0016, P = 0.9681 | No |

*Behaviours*

| **Behaviour** | **Never smoked mean** | **Smoking history mean** | **Analysis** | **Significant** |
| --- | --- | --- | --- | --- |
| Ask | 4.000 | 4.0256 | F(1,108)=0.0099, p = 0.9207 | No |
| Record | 2.6250 | 3.0641 | F(1,108)=1.4486, p = 0.2314 | No |
| Inform | 3.2500 | 3.6667 | F(1,108)=2.072, p = 0.1529 | No |
| Brief advice | 3.5313 | 3.5769 | F(1,108)=0.031, p = 0.8606 | No |
| Refer | 3.1250 | 3.2692 | F(1,108)=0.2671, p = 0.6063 | No |
| NRT | 3.1875 | 3.3718 | F(1,108)=0.3665, p = 0.5462 | No |

*TDF Domains*

| **Domain** | **Never smoked mean** | **Smoking history mean** | **Analysis** | **Significant** |
| --- | --- | --- | --- | --- |
| Knowledge | 3.8705 | 3.9249 | F(1,108)=0.1535, p = 0.6959 | No |
| Skills | 3.0000 | 3.0513 | F(1,108)=0.2248, p = 0.6364 | No |
| Soc/prof role | 3.8281 | 4.0513 | F(1,108)=1.347, p = 0.2484 | No |
| Beliefs cap | 3.3984 | 3.3526 | F(1,108)=0.2276, p = 0.6343 | No |
| Optimism | 4.0937 | 4.2692 | F(1,108)=1.1779, p = 0.2802 | No |
| Beliefs cons | 3.6562 | 3.7735 | F(1.108)=0.7736, p = 0.381 | No |
| Reinforcement | 3.0312 | 3.4103 | F(1,108)=3.5258, p = 0.0631 | Weak evidence p<0.10 |
| Intentions | 4.0625 | 4.0641 | F(1,108)=0.0001, p = 0.9928 | No |
| Goals | 3.2969 | 3.4423 | F(1,108)=0.706, p = 0.4026 | No |
| Memory | 3.3594 | 3.4167 | F(1,108)=0.0818, p = 0.7754 | No |
| Environment | 3.3229 | 3.2607 | F(1,108)=0.1112, p = 0.7394 | No |
| Social influences | 3.1094 | 3.2276 | F(1,108)=1.4254, p = 0.2351 | No |
| Emotion | 3.8437 | 3.5769 | F(1,108)= 1.7578, p = 0.1877 | No |
| Beh regulation | 3.4844 | 3.4679 | F(1,108)= 0.0076, p = 0.9309 | No |

1. **Training experience**

*Perceived importance*

| **IMPORTANCE RATING** | **No Training mean** | **Training mean** | **Analysis** | **Significant** |
| --- | --- | --- | --- | --- |
|  | 3.9355 | 4.1923 | F(1,107)=1.5978, p = 0.209 | No |

*Behaviours*

| **Behaviour** | **No Training** | **Training** | **Analysis** | **Significant** |
| --- | --- | --- | --- | --- |
| Ask | 4.0323 | 4.0127 | F(1,108)=0.0057, p = 0.9399 | No |
| Record | 2.8065 | 2.9873 | F(1,108)=0.2385, p = 0.6263 | No |
| Inform | 3.3226 | 3.6329 | F(1,108)=1.118, p = 0.2927 | No |
| Brief advice | 3.1935 | 3.7089 | F(1,108)=4.0121, p = 0.0477 | Yes |
| Refer | 2.8387 | 3.3797 | F(1,108)=3.8086, p = 0.0536 | Weak |
| NRT | 3.0968 | 3.4051 | F(1,108)=1.0123, p = 0.3166 | No |

*TDF Domains (note order in table flipped from above)*

| **Domain** | **Training mean** | **No training mean** | **Analysis** | **Significant** |
| --- | --- | --- | --- | --- |
| Knowledge | 4.0145 | 3.6406 | F(1,108)=7.6158, p = 0.0068 | Yes |
| Skills | 3.1741 | 2.6855 | F(1,108)=24.5114, p = 0 | Yes |
| Soc/prof role | 4.0506 | 3.8226 | F(1,108)=1.3807, p = 0.2426 | No |
| Beliefs cap | 3.4304 | 3.2016 | F(1,108)=5.8408, p = 0.0173 | Yes |
| Optimism | 4.2658 | 4.0968 | F(1,108)=1.0715, p = 0.3029 | No |
| Beliefs cons | 3.7806 | 3.6344 | F91,108)=1.1843, p = 0.2789 | No |
| Reinforcement | 3.4684 | 2.8710 | F(1,108)=9.0178, p = 0.0033 | Yes |
| Intentions | 4.1266 | 3.9032 | F(1,108)=1.59, p = 0.21 | No |
| Goals | 3.5253 | 3.0806 | F(1,108)=6.8411, p = 0.0102 | Yes |
| Memory | 3.5506 | 3.0161 | F(1,108)=7.4636, p = 0.0074 | Yes |
| Environment | 3.4135 | 2.9355 | F(1,108)=6.8368, p = 0.0102 | Yes |
| Social influences | 3.269 | 3.000 | F(1,108)=7.6568, p = 0.0067 | Yes |
| Emotion | 3.7215 | 3.4839 | F(1,108)=1.3632, p = 0.2456 | No |
| Beh regulation | 3.5696 | 3.2258 | F(1,108)=3.3523, p = 0.0699 | Weak evidence p<0.10 |

1. **Pay band**

*Perceived importance*

| **IMPORTANCE RATING** | **Band 5 mean** | **Band 6 mean** | **Band 7 mean** | **Band 8 mean** | **Analysis** | **Significant** |
| --- | --- | --- | --- | --- | --- | --- |
|  | 4.0256 | 4.3913 | 4.0270 | 4.2000 | F(1,105)=0.8744, p = 0.4569 | No |

*Behaviours*

| **Behaviour** | **Band 5 mean** | **Band 6 mean** | **Band 7 mean** | **Band 8 mean** | **Analysis** | **Significant** |
| --- | --- | --- | --- | --- | --- | --- |
| Ask | 4.0000 | 4.2917 | 3.9730 | 3.6000 | F(3,106)=0.8106, p = 0.4907 | No |
| Record | 3.5897 | 3.4583 | 2.2703 | 1.6000 | F(3,106)=7.4347, p<0.001 | Yes, significant differences between pay bands:  5-7, 5-8, 6-7, 6-8 |
| Inform | 4.1026 | 4.000 | 2.8919 | 2.7000 | F(3,106)=8.3534, p <0.001 | Yes, significant differences between pay bands:  5-7, 5-8, 6-7, 6-8 |
| Brief advice | 3.6154 | 3.8750 | 3.4054 | 3.2000 | F(3,106)=1.0311, p = 0.382 | No |
| Refer | 3.2821 | 3.5417 | 3.2973 | 2.000 | F(3,106)=3.6095, p = 0.0158 | Yes, significant differences between pay bands:  5-8, 6-8, 7-8 |
| NRT | 3.6154 | 3.5000 | 3.1351 | 2.4000 | F(3,106)=2.2969, p = 0.0818 | Weak, significant differences between pay bands:  5-8, 6-8 |

*TDF Domains*

| **Domain** | **Band 5 mean** | **Band 6 mean** | **Band 7 mean** | **Band 8 mean** | **Analysis** | **Significant** |
| --- | --- | --- | --- | --- | --- | --- |
| Knowledge | 3.8462 | 4.0357 | 3.9305 | 3.7714 | F(3,106)=0.5666, p = 0.6382 | No |
| Skills | 2.9551 | 3.0729 | 3.1419 | 2.8750 | F(3,106)=1.2239, p = 0.3047 | No |
| Soc/prof role | 3.9872 | 4.2083 | 3.9189 | 3.7000 | F(3,106)=0.8565, p = 0.4662 | No |
| Beliefs cap | 3.3205 | 3.4896 | 3.3378 | 3.3500 | F(3,106)=0.7616, p = 0.5181 | No |
| Optimism | 4.1026 | 4.4167 | 4.2432 | 4.1000 | F(3,106)=0.9121, p = 0.4378 | No |
| Beliefs cons | 3.7863 | 3.9306 | 3.6486 | 3.4333 | F(3, 106)= 1.8697, p = 0.1392 | No |
| Reinforcement | 3.3590 | 3.5000 | 3.2162 | 2.9000 | F(3,106)=1.0427, p= 0.3769 | No |
| Intentions | 4.0256 | 4.2083 | 4.0541 | 3.9000 | F(3,106)=0.3873, p = 0.7624 | No |
| Goals | 3.4615 | 3.3125 | 3.4730 | 3.1000 | F(3,106)=0.6964, p = 0.5562 | No |
| Memory | 3.4103 | 3.6250 | 3.4054 | 2.8000 | F(3,106)=1.82, p = 0.148 | No |
| Environment | 3.2564 | 3.3889 | 3.2703 | 3.1333 | F(3,106)=0.2183, p = 0.8835 | No |
| Social influences | 3.1410 | 3.1667 | 3.2770 | 3.1500 | F(3,106)= 0.5931, p= 0.6208 | No |
| Emotion | 3.4615 | 3.7500 | 3.7297 | 3.9000 | F(3,106)=0.8917, p= 0.448 | No |
| Beh regulation | 3.4231 | 3.6667 | 3.5676 | 2.8500 | F(3,106)=2.2391, p = 0.088 | Weak evidence  Significant differences between band 8 and other pay bands:  Band 5 – Band 8: p=0.0692  Band 6 – Band 8: p=0.0154  Band 7 – Band 8: p=0.0242 |
